# Sphingosine 1-phosphate receptor 5 mediates the immune quiescence of the human brain endothelial barrier

**DOI:** 10.1186/1742-2094-9-133

**Published:** 2012-06-20

**Authors:** Ruben van Doorn, Melissa A Lopes Pinheiro, Gijs Kooij, Kim Lakeman, Bert van het Hof, Susanne MA van der Pol, Dirk Geerts, Jack van Horssen, Paul van der Valk, Elizabeth van der Kam, Eric Ronken, Arie Reijerkerk, Helga E de Vries

**Affiliations:** 1 Department of Molecular Cell Biology and Immunology, VU University Medical Center, , P.O. Box 7057, 1007 MB, Amsterdam, The Netherlands; 2 Department of Pediatric Oncology/Hematology, Sophia Children’s Hospital, Erasmus University Medical Center, Dr. Molewaterplein 60, 3015 GJ, Rotterdam, The Netherlands; 3 Department of Pharmacology, Abbott GmbH & Co KG, Knollstrasse 50, Ludwigshafen, 67061, Germany; 4 Department of Pathology, VU University Medical Center, De Boelelaan 1117, 1081 HV, Amsterdam, The Netherlands; 5 Spinoza Centre for Neuroimaging, Royal Netherlands Academy of Arts and Sciences Netherlands Institute for Neuroscience (NIN), Meibergdreef 47, 1105BA, Amsterdam, The Netherlands

**Keywords:** Sphingosine 1-phosphate (S1P) receptor, FTY720P, Blood–brain barrier, Neuroinflammation, Monocyte, Multiple sclerosis

## Abstract

**Background:**

The sphingosine 1-phosphate (S1P) receptor modulator FTY720P (Gilenya®) potently reduces relapse rate and lesion activity in the neuroinflammatory disorder multiple sclerosis. Although most of its efficacy has been shown to be related to immunosuppression through the induction of lymphopenia, it has been suggested that a number of its beneficial effects are related to altered endothelial and blood–brain barrier (BBB) functionality. However, to date it remains unknown whether brain endothelial S1P receptors are involved in the maintenance of the function of the BBB thereby mediating immune quiescence of the brain. Here we demonstrate that the brain endothelial receptor S1P_5_ largely contributes to the maintenance of brain endothelial barrier function.

**Methods:**

We analyzed the expression of S1P_5_ in human post-mortem tissues using immunohistochemistry. The function of S1P_5_ at the BBB was assessed in cultured human brain endothelial cells (ECs) using agonists and lentivirus-mediated knockdown of S1P_5_. Subsequent analyses of different aspects of the brain EC barrier included the formation of a tight barrier, the expression of BBB proteins and markers of inflammation and monocyte transmigration.

**Results:**

We show that activation of S1P_5_ on cultured human brain ECs by a selective agonist elicits enhanced barrier integrity and reduced transendothelial migration of monocytes *in vitro*. These results were corroborated by genetically silencing S1P_5_ in brain ECs. Interestingly, functional studies with these cells revealed that S1P_5_ strongly contributes to brain EC barrier function and underlies the expression of specific BBB endothelial characteristics such as tight junctions and permeability. In addition, S1P_5_ maintains the immunoquiescent state of brain ECs with low expression levels of leukocyte adhesion molecules and inflammatory chemokines and cytokines through lowering the activation of the transcription factor NFκB.

**Conclusion:**

Our findings demonstrate that S1P_5_ in brain ECs contributes to optimal barrier formation and maintenance of immune quiescence of the barrier endothelium.

## Background

The vasculature of the brain is specialized to function as a barrier to protect the central nervous system (CNS) by restricting entry of unwanted molecules and immune cells into the brain. This so-called blood–brain barrier (BBB) is composed of highly specialized brain endothelial cells (ECs) that line the capillary wall. These specialized ECs form a tight barrier by membrane efflux pumps that drive cellular exclusion of unwanted compounds and the expression of complex tight junctions [1-4], which actively limit cellular infiltration into the brain. The ECs are enclosed together with pericytes within the basement membrane onto which astrocytes firmly project their endfeet, thereby maintaining the barrier properties within the endothelium [5]. Strikingly, several neuroinflammatory and neurodegenerative diseases such as multiple sclerosis (MS), human immunodeficiency virus-associated dementia, capillary cerebral amyloid angiopathy and stroke are associated with an impaired function of the BBB [6-9]. Especially in MS, an altered BBB function leads to enhanced entry of immune cells and potentially toxic compounds into the CNS [10-12]. To date, it remains largely unknown which mechanisms underlie the altered BBB in such neurological disorders. The identification of targets to restore impaired barrier function may provide novel tools for treatment.

Sphingosine 1-phosphate (S1P) binding G-protein-coupled receptors (GPCRs) are thought to be involved in the regulation of the vasculature. In general, S1P signals through five GPCRs belonging to the endothelial differentiation gene (EDG) family which entails: S1P_1_ (EDG-1), S1P_2_ (EDG-5), S1P_3_ (EDG-3), S1P_4_ (EDG-6), and S1P_5_ (EDG-8). The S1P receptors are coupled to different G-proteins resulting in divergent downstream signaling pathways. For example, S1P_1_ is proposed to couple to Gα_i_ and Gα_o_ whereas S1P_5_ is through to interact with Gα_i_, Gα_o_, Gα_12_, and Gα_13_[13]. Consequently, signaling of S1P through its receptors affects a broad variety of signaling processes ranging from pathways involved in cell survival, proliferation, motility to differentiation. S1P receptors are essential in proper vascular development because deficiency of for example S1P_1_ results in early embryonic death due to defects in vascular maturation [14]. Moreover, numerous S1P-driven EC responses are reported and are found to be crucial for proper development, maintenance and regulation of peripheral vascular beds. S1P-mediated effects on the function of the endothelium are attributed to modulation of S1P_1_ and S1P_3_. S1P receptors are currently under extensive investigation because clinical trials demonstrate significant reduction of disease severity in neurological (autoimmune) disease models by targeting these receptors [15-19]. In addition, two phase III clinical trials (TRANSFORMS, FREEDOMS) concerning the S1P receptor modulator FTY720P (Gilenya®) for treatment of relapsing remitting MS deliver robust data demonstrating greatly reduced relapse rates, significant reduction in lesion activity, and lower risk of disability progression [20,21].

The recent availability of non-selective and selective S1P receptor agonists has led to an increasing amount of data about S1P receptor expression and their impact on cellular function in the CNS. Apparent CNS effects of S1P receptor modulators such as FTY720P and S1P itself are translated into for example reduced immune cell infiltration across the brain vasculature [19,22-24]. Recently, we demonstrated increased astrocytic S1P_1_ and S1P_3_ expression in active MS lesions and an anti-inflammatory effect of FTY720P (active form of FTY720) on primary human astrocyte cultures [25]. Interestingly, although S1P_1-4_ receptors are widely expressed throughout the body, S1P_5_ expression is more or less restricted to the brain. It was therefore the aim of this study to investigate the involvement of S1P_5_ in underlying inflammatory processes in the CNS leading to MS lesion development. We here show that S1P_5_ is primarily expressed on brain ECs in human brain suggesting a key role of S1P_5_ in BBB maintenance. Our functional studies show that S1P_5_ is not only crucial for the maintenance of the BBB but is also a key modulator of endothelial inflammation processes. Ultimately, specific targeting of vascular S1P_5_ and subsequent repair of the BBB in patients with inflammatory disorders may have therapeutic benefits.

## Methods

### Autopsy material

Brain tissue from three non-neurological controls (Table 1) was obtained at rapid autopsy and immediately frozen in liquid nitrogen or fixed in formalin (in collaboration with The Netherlands Brain Bank, coordinator Dr. Huitinga). The Netherlands Brain Bank received permission to perform autopsies for the use of tissue and for access to medical records for research purposes from the Ethical Committee of the VU University Medical Center, Amsterdam, The Netherlands. Tissue samples from control cases without neurological disease were taken from the subcortical white matter. All controls, or their next of kin, had given informed consent for autopsy and the use of their brain tissue for research purposes.

**Table 1:** Clinical data of non-neurological controls

**Case**	**Age (years)**	**Type of MS**	**Sex**	**Post-mortem** **delay (h:min)**	**Disease** **Duration** **(years)**	**Cause of death**
Control	89	NA	F	6	NA	Old age
Control	89	NA	M	16	NA	Dehydration
Control	84	NA	F	5	NA	Resp. failure

NA = not applicable; m = male; f = female.

### Immunohistochemistry

For immunohistochemical analysis, 5-μm cryosections were processed and stained as described previously [26]. Briefly, sections were incubated overnight at 4 °C with primary antibodies against S1P_5_ (1:200) (Santa Cruz Biotechnology, Santa Cruz, CA, USA and Imgenex, San Diego, CA, USA). Subsequently, sections were incubated with EnVision + Dual Link (DAKO, Glostrup, Denmark) according to the manufacturer’s description. Diaminobenzidine tetrachloride (DAB; DAKO, Glostrup, Denmark) was used as the chromogen. Antibodies were diluted in PBS containing 0.1% bovine serum albumin (BSA; Boehringer Mannheim, Mannheim, Germany), which also served as a negative control. For colocalization studies, sections were incubated for 30 minutes with 5% goat serum. Subsequently, sections were incubated with primary antibodies as described above and with mouse anti-CD31 (1:200; DAKO, Glostrup, Denmark). Sections were then incubated with goat anti-mouse Alexa 488 and goat anti-rabbit Alexa 594 secondary antibodies (1:400 Molecular Probes, Eugene OR, USA). Omission of primary antibodies served as a negative control. Incubation of tissue sections with isotype controls or no secondary antibody showed no immunoreactivity.

### Endothelial cell culture

The human brain EC line hCMEC/D3 was kindly provided by Prof. P-O. Couraud (Institut Cochin, Université Paris Descartes, Paris, France) [27] and grown in Endothelial Cell Basal Medium-2 supplemented with hEGF, hydrocortisone, GA-1000, FBS, VEGF, hFGF-B, R^3^-IGF-1, ascorbic acid and 2.5% fetal calf serum (FCS; EGM-2, Lonza, Basel, Switzerland). Cells were cultured as described before [28].

### Lentiviral shRNA for S1P5 knockdown

Selective gene knockdown was obtained by using a vector-based short hairpin (sh) RNA technique as described before [29]. Plasmids encoding S1P_5_-specific shRNAs were obtained from Sigma (TRCN0000004752, St Louis, MO, USA). Recombinant lentiviruses were produced by co-transfecting subconfluent HEK 293 T cells with the specific expression plasmids and packaging plasmids (pMDLg/pRRE, pRSV-Rev and pMD2G) using calcium phosphate as a transfection reagent. HEK 293 T cells were cultured in DMEM supplemented with 10% FCS, 1% penicillin/streptomycin, in a 37 °C incubator with 5% CO_2_. Infectious lentiviruses were collected 48 hours after transfection and stored at −80 °C. Subsequently, lentiviruses expressing S1P_5_-specific shRNA were used to transduce hCMEC/D3 cells. Control cells were generated by transduction with lentivirus expressing non-targeting shRNA (SHC002, Sigma, St Louis, MO, USA). Forty-eight hours after infection of hCMEC/D3 cells with the shRNA-expressing lentiviruses, stable cell lines were selected by puromycin treatment (2 μg/ml). The expression knockdown efficiency was determined by quantitative PCR (qPCR).

### Quantitative PCR

All oligonucleotides were synthesized by Ocimum Biosolutions (Ocimum Biosolutions, Ijsselstein, The Netherlands) (Table 2), RNA was isolated using the Aurum^TM^ Total RNA capture kit (Biorad, CA, USA) according to the manufacturer’s instructions. cDNA was synthesized with the Reverse Transcription System kit (Promega, Madison, WI, USA) following manufacturer’s guidelines as described previously [30]. qPCR reactions were performed in an ABI7900HT sequence detection system with the SYBR Green method (Applied Biosystems, Carlsbad, CA, USA). Obtained expression levels of transcripts were normalized to GAPDH expression levels.

**Table 2:** Primers

**Primer**	**Sequence Forward**	**Sequence Reverse**
S1P_1_	5′-TGCGGGAAGGGAGTATGTTT-3′	5′-CGATGGCGAGGAGACTGAAC-3′
S1P_2_	5′-TCTCTACGCCAAGCATTATGTGC-3′	5′-TGGCCAACAGGATGATGGA-3′
S1P_3_	5′-TGCAGCTTCATCGTCTTGGAG-3′	5′-GCCAATGAAAAAGTACATGCGG-3′
S1P_5_	5′-CCTTGGTGGCATGTTGGG-3′	5′-GGGTTCAGAAGTGAGTTGGG-3′
GLUT	5′-GCCCCTGTGAAGATTGAGAG-3′	5′-CCCGAAGCAGCCAATCC-3′
BCRP	5′-AGATGGGTTTCCAAGCGTTCAT-3′	5′-CCAGTCCCAGTACGACTGTGACA-3′
PGP	5′-GTCCCAGGAGCCCATCCT-3′	5′-CCCGGCTGTTGTCTCCATA-3′
VE-cadherin	5′-TGACGTGAACGACAACTGGC-3′	5′-GACGCATTGAACAACCGATG-3′
Claudin-5	5′-GCCCCTGTGAAGATTGAGAG-3′	5′-CCCGAAGCAGCCAATCC-3′
TNF-α	5′-CCAAGCCCTGGTATGAGCC-3′	5′-GCCGATTGATCTCAGCGC-3′
IL-1β	5′-GCTGATGGCCCTAAACAGATG-3′	5′- GCAGAGGTCCAGGTCCTGG-3′
IL-8	5′-TGAGAGTGGACCACACTGCG-3′	5′-TCTCCACAACCCTCTGCACC-3′
MCP-1	5′-ATCTCAGTGCAGAGGCTCGC-3′	5′-GCACAGATCTCCTTGGCCAC-3′
VCAM	5′-TGAAGGATGCGGGAGTATATGA-3	′5′-TTAAGGAGGATGCAAAATAGAGCA-3
ICAM	5′-TAGCAGCCGCAGTCATAATGGG-3′	′5′-AGGCGTGGCTTGTGTGTTCG-3

### Western blot

Cell homogenates were prepared by replacing the culture medium with sodium dodecyl sulfate (SDS) sample buffer containing 5% ß-mercaptoethanol and subsequent heating at 95 °C for 5 minutes. Samples were separated by SDS-PAGE and blotted onto nitrocellulose membranes. Membranes were blocked with Odyssey block buffer and incubated with an antibody against S1P_5_ (Santa Cruz Biotechnology, Santa Cruz, CA, USA). Binding was visualized using the Odyssey® Infrared Imaging System after application of IgG labeled with Infrared dye (IRdye) 800 (Rockland Immunochemicals, Gilbertsville, PA, USA). For quantification, S1P_5__._protein levels were corrected for tubulin using a specific anti-tubulin antibody (Cedarlane, Canada). The basal expression of p65 in S1P_5_ knockdown and control cells was assessed with a rabbit anti-p65 antibody (Cell Signaling Technology, Danvers, MA, USA) followed by secondary donkey anti-rabbit 800 (IRDye). Protein expression was normalized to beta-actin (Santa Cruz Biotechnology, Santa Cruz, CA, USA) detected with donkey anti-goat 680 (IRDye).

### Immunocytochemistry

Transduced (mock and S1P_5_) hCMEC/D3 cells were plated in collagen coated μ-slide 8 well slides (Ibidi, Martinsried, Germany) and cultured as described before. Upon confluence, cells were cultured in Endothelial Cell Basal Medium-2 containing 2,5% human serum and 5 ng/ml basic fibroblast growth factor (bFGF) overnight and fixed in 4% formaldehyde (Sigma, St Louis, MO, USA) in PBS (Gibco) in the presence of 0.5% Triton (Sigma, St Louis, MO, USA). Cells were washed and blocked with PBS containing 0.1% BSA (Sigma, St Louis, MO, USA) and 5% normal goat serum (NGS). Subsequently, cells were incubated with mouse anti-VE-cadherin primary antibody (1:500: Becton & Dickinson, San Jose, CA, USA) in PBS containing 0.1% BSA and 5% NGS overnight. Cells were washed and incubated with goat anti-mouse Alexa 488 secondary antibody (1:400: Molecular Probes, Eugene OR, USA). After washing the cells with PBS, rhodamine phalloidin (1:300) (Molecular Probes, Eugene OR, USA) was used for F-actin staining and Hoechst (1:1000 Molecular Probes, Eugene OR, USA) for nuclear staining.

### Electrical cell-substrate impedance sensing

Transendothelial electric resistance in confluent monolayers of hCMEC/D3 cells was measured using an electrical cell-substrate impedance sensing (ECIS) model ZTheta (Applied BioPhysics, NY, USA) and 8W10E + arrays. In short, 300 μl cell suspension (1,0 × 10^5^ cells) was added to in each well in Endothelial Cell Basal Medium-2 supplemented with 2,5% human serum and 5 ηg/ml bFGF. Cells were seeded in medium containing dimethyl sulfoxide (DMSO; 1 μM serving as vehicle control), FTY720P (1 μM), or a S1P_5_ selective agonist (1 μM compound 18, a kind gift from Professor Stephen Hanessian, Université de Montreal, Montreal, Canada) [31]. Prior to seeding the collagen-coated ECIS arrays were equilibrated with growth medium and Rb values were calculated using ECIS software version 1.2.55.0 PC.

### Permeability of the brain endothelial layer

Permeability of human brain EC monolayers was analyzed as described previously [28]. hCMEC/D3 and hCMEC/D3 S1P_5_ knockdown cells were seeded at confluence onto collagen-coated Costar Transwell filter (pore-size 0.4 μm; Corning Incorporated, Corning, NY, USA) in growth medium containing 2.5% FCS and grown for 4 days. Paracellular permeability to FITC-dextran (150 kDa, 500 μg/ml in culture medium; Sigma, St Louis, MO, USA-Aldrich) in the apical to basolateral direction was assayed at various time points. Samples were collected from the acceptor chambers for measurement of fluorescence intensity using a FLUOstar Galaxy microplate reader (BMG Labtechnologies, Offenburg, Germany), excitation 485 nm and emission 520 nm.

### Monocyte migration

Human blood monocytes were isolated from buffy coats of healthy donors (Sanquin, Blood Bank, Amsterdam, NL) by Ficoll gradient and CD14-positive beads [32]. Control and S1P_5_ knockdown hCMEC/D3 cells were seeded at confluence onto collagen-coated Costar Transwell filters (pore-size 5 μm; Corning Incorporated, Corning, NY, USA) in growth medium containing 2.5% FCS and were grown for 4 days. ECs were exposed to DMSO (1 μM) as a vehicle control, FTY720P (1 μM) or S1P_5_ agonist (1 μM) agonist for 16 hours and washed prior to addition of monocytes [31]. Hereafter, 100 μl 1.6 × 10^6^/ml primary human monocytes suspended in Endothelial cell Growth medium-2 containing 2,5% FCS were added to the upper compartment for 8 hours at 37°C, 5% CO_2_ in air. Next, the suspension in the lower compartment containing transmigrated monocytes was harvested and quantified using anti-CD14 beads (Flow-count fluorospheres, BeckmanCoulter, Inc., Brea, CA, USA) and subsequent FACScan flow cytometer (Becton & Dickinson, San Jose, CA, USA) analyses.

### Monocyte adhesion assay

Monocyte adhesion to confluent monolayers of control and S1P5 knockdown hCMEC/D3 cells was analyzed by using primary human monocytes isolated as described before. Human monocytes were fluorescently labeled with 0.5 μM calcein-Am (Molecular Probes, Eugene OR, USA) and suspended in RPMI + 0.5% BSA + 25 mM Hepes to a final concentration of 1 × 10^6^ cells [33]. A standard curve of human monocytes was made with 0, 12.5, 25, 50, and 100% of this cell suspension. ECs were washed and monocytes were added and incubated for 5, 15, 30, and 60 minutes in a 37 °C incubator with 5% CO_2_. Non-adherent cells were removed and adherent cells were lysed with 0.1 M NaOH. Fluorescence intensity was measured (FLUOstar Galaxy, BMG Labtechnologies, Offenburg, Germany; excitation 480 nm, emission 520 nm) and the number of adhered monocytes was calculated using the standard [33].

### Flow cytometric analysis

Control and S1P_5_ knockdown hCMEC/D3 cells were detached from 24-well culture plates, washed and incubated with monoclonal mouse anti-intercellular adhesion molecule-1(ICAM-1) (Rek-1, 5 μg/ml, a kind gift from the Department of Tumour Immunology, University Medical Center St. Radboud, Nijmegen, The Netherlands) or mouse anti-vascular cell adhesion molecule-1(VCAM-1; AbD Serotec, UK) for 30 minutes at 4 °C [34]. Binding was detected using goat anti-mouse Alexa 488 (Molecular Probes, Eugene, OR). Omission of primary antibodies served as negative control. To investigate the role of Nuclear factor kappa-light-chain-enhancer of activated B cells (NFκB) in the enhanced inflammatory status of S1P_5_ knockdown cells, the cells were treated for 16 hours with 4 μM NFκB inhibitor Bay 11–7085 (Sigma, MO, USA) or vehicle control. Fluorescence intensity was measured using a FACS Calibur flow cytometer (Becton & Dickinson, San Jose, CA, USA). The mean fluorescence intensity was used as a measure for the expression of ICAM-1 and VCAM-1.

### Statistical analysis

Data are presented as ± SEM and were analyzed statistically by means of single-column *t*-test. Statistical significance was defined as *P <0.05, **P <0.002, and ***P <0.001.

## Results

### S1P_5_ is highly expressed by brain capillaries in the human brain

**Figure 1:**
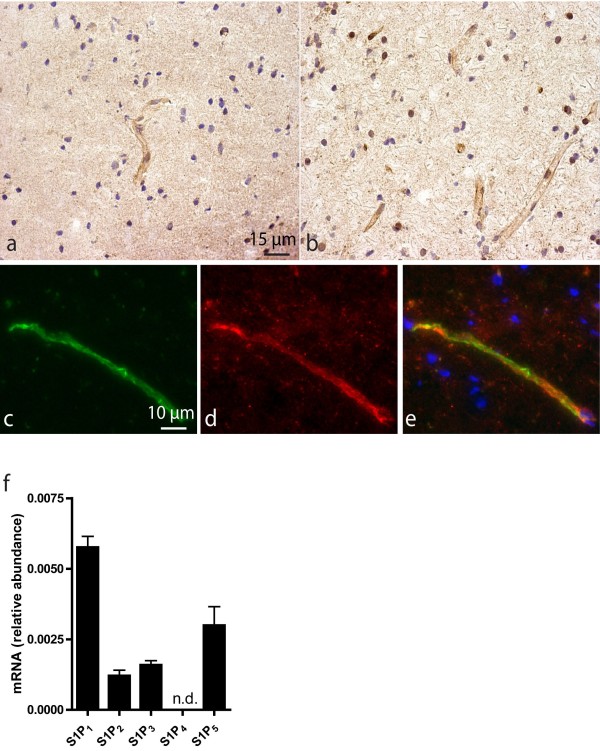
**S1P**_**5**_**is expressed in the human cerebrovasculature.** Capillaries in white matter from non-neurological human controls show strong S1P_5_ expression. Human S1P_5_ expression was determined with either the Santa Cruz (**a**) or the Imgenex (**b**) antibody both showing similar staining patterns. Colocalization studies with the EC marker CD31 (**c**) confirmed that ECs express significant levels of S1P_5_ (**d**) in human brain capillaries (**e**). S1P receptor RNA expression levels of hCMEC/D3 cells were determined by qPCR. S1P_1_ expression was most abundant while there was no detectable level of S1P_4_ expression. Receptor expression was detected in the following order: S1P_1_ > S1P_5_ > S1P_3_ > S1P_2_ where S1P_1_ expression level was set at 100% (**f**). RNA encoding for S1P4 was undetectable (n.d.). Data represent ± SEM of three independent experiments.

The expression of the S1P_5_ was analyzed in the white matter of non-neurological control patients. Immunohistochemical staining with the two different anti-S1P_5_ antibodies, clone H-88 (Santa Cruz) (Figure 1a) and IMG-71372 (Imgenex) (Figure 1b), essentially yield identical results. Both antibodies revealed that human brain capillaries in the white and the gray matter (not shown) constitutively express S1P_5_. Colocalization studies using the endothelial marker CD31 (PECAM-1; Figure 1c) together with antibodies against S1P_5_ (Figure 1d) identified ECs as the most predominant S1P_5_ expressing cell type within the human brain (Figure 1e). Gene expression analysis of the different S1P receptors in hCMEC/D3 cells indicated that brain ECs express all S1P receptors except S1P_4_ at different levels (S1P_1_ > S1P_5_ > S1P_3_ > S1P_2_ with S1P_1_ being the highest; Figure 1f).

### FTY720P and a selective S1P_5_ agonist enhance transendothelial electrical resistance

**Figure 2:**
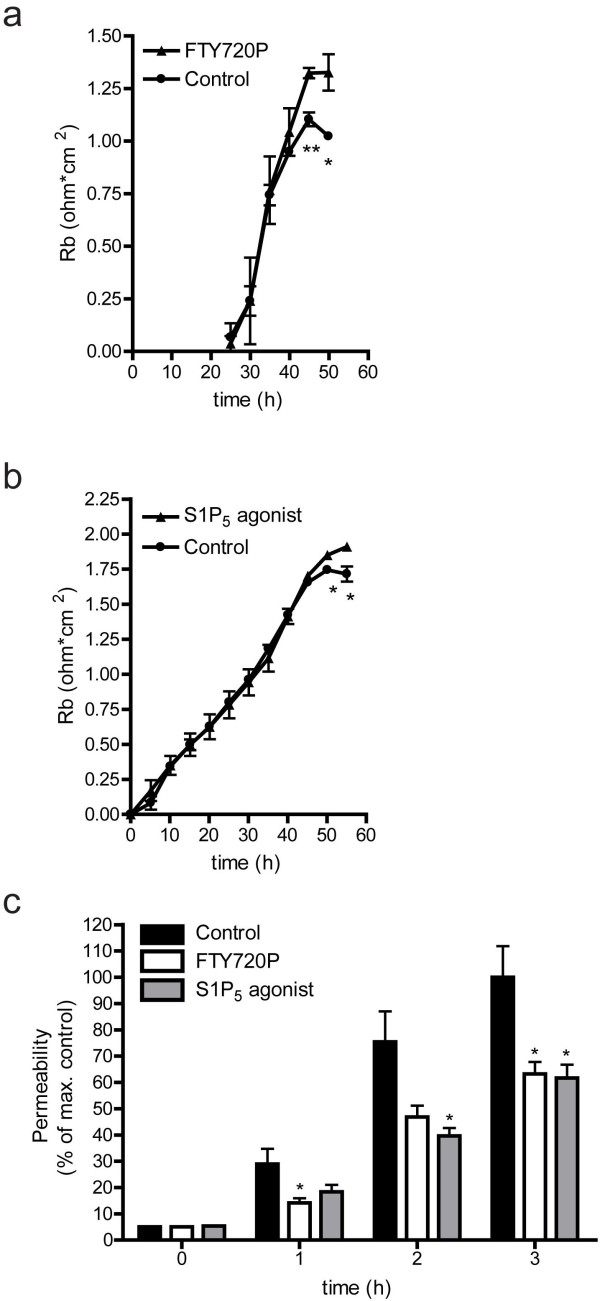
**S1P**_**5**_**activation enhances barrier endothelial integrity.** S1P receptor modulators were used to determine their effect of the transendothelial electrical resistance (TEER) as assayed through ECIS. (**a**) The non-selective S1P receptor modulator FTY720P (10^−6^ M in DMSO) enhanced the TEER of hCMEC/D3 cells to 1.32 ohm.cm^2^ ± 0.02, n = 3 compared to vehicle control at final time point (1.10 ohm.cm^2^ ± 0.03, n = 3). Statistical significance (*t*-test) is indicated with asterisks: *P < 0.05 and **P < 0.005. (**b**) The selective S1P_5_ agonist (10^−6^ M in DMSO) enhanced TEER of hCMEC/D3 cells to 1.91 ohm.cm^2^ ± 0.03, n = 4 at final time point compared to vehicle control (1.72 ohm.cm^2^ ± 0.05, n = 4) (B). The Rb values represent the mean ± SEM of four independent experiments. Statistical significance (*t*-test) is indicated with asterisks: *P < 0.05. (**c**) Both the non-selective S1P receptor modulator FTY720P (10^−6^ M in DMSO) and the selective S1P_5_ agonist (10^−6^ M in DMSO) reduced permeability of hCMEC/D3 cells for FITC-dextran (FD 70) by 63.2% ± 4.6 (n = 4) and 61.7% ± 5.0 (n = 4), respectively. Fluorescence intensity values represent mean ± SEM of four individual experiments with hCMEC/D3 value at 5hours set at 100% (100.0% ± 11.8, n = 4). Statistical significance (*t*-test) is indicated with asterisks: *P < 0.05.

To investigate the role of S1P_5_ in brain EC barrier function, we measured paracellular resistance formation by hCMEC/D3 cells while exposed to either FTY720P or the selective S1P_5_ agonist [31] by means of ECIS. Our results show that S1P receptor activation by the non-selective S1P receptor agonist FTY720P significantly increases barrier formation in comparison with control cells (Figure 2a). At the same time, stimulation of the brain ECs with the selective S1P_5_ agonist also significantly enhanced barrier formation in comparison to controls (Figure 2b), indicating that S1P_5_ agonism modulates barrier formation. Moreover, treatment of brain endothelial monolayers with both the non-selective S1P receptor modulator FTY720P and the selective S1P_5_ agonist significantly reduced permeability of hCMEC/D3 cells for FITC-dextran (70kD) by 63.2% ± 4.6 and 61.7 ± 5.0, respectively (Figure 2c).

### S1P agonism reduces transendothelial migration of monocytes

**Figure 3:**
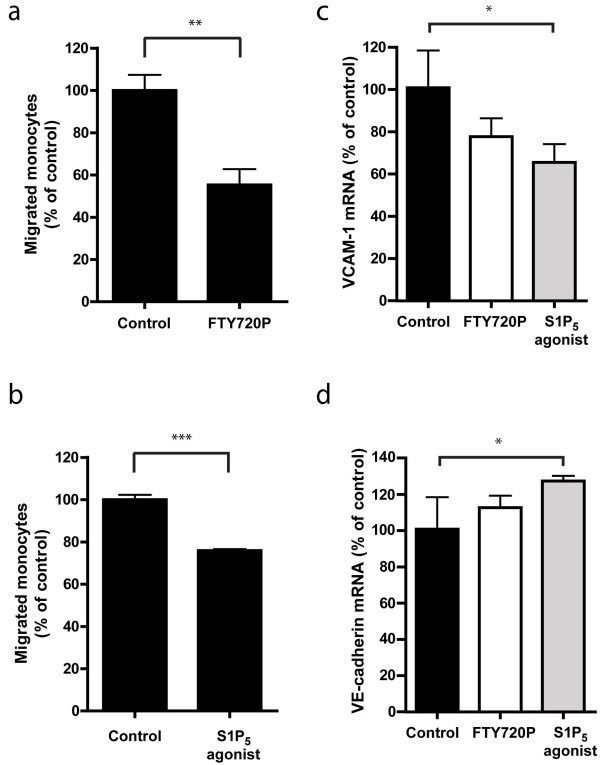
**Endothelial S1P**_**5**_**activation decreases monocyte transmigration.** FTY720P (10^−6^ M in DMSO; **a**) and a S1P_5_ agonist (10^−6^ M in DMSO; **b**) were added to confluent monolayers of hCMEC/D3 cells for 24 hours. Monocyte migration was assayed by time lapse microscopy, FTY720P treatment of the brain ECs reduced monocyte migration to 55.3% ± 7.42 (n = 3) compared to control (106.0% ± 6.14 n = 3; **a**) and treatment with the selective S1P_5_ agonist reduced monocyte migration to 75.9% ± 0.68 n = 3; **b**). Both FTY720P and S1P_5_ agonist treated cells show reduced VCAM-1 expression (control: 100.0% ± 17.0; S1P_5_ agonist 64.8% ± 8.9, n = 3; **c**) and increased VE-cadherin expression (control: 100.0% ± 8.5; S1P_5_ agonist: 126.3% ± 2. 6, n = 3; **d**). Values represent the mean ± SEM of three individual experiments each performed in triplicate with controls set at 100%. Statistical significance (*t*-test) is indicated with asterisks: *P < 0.05 **P < 0.002 and ***P < 0.001.

We next studied the effect of FTY720P and the selective S1P_5_ agonist on a hallmark of neuroinflammation, namely monocyte migration across the brain EC barrier. hCMEC/D3 cells were exposed to FTY720P or the selective S1P_5_ agonist for 24 hours prior to the addition of primary human monocytes. In concordance with the results described above, treatment with both FTY720P (Figure 3a) and the selective S1P_5_ agonist (Figure 3b) resulted in reduced transmigration of monocytes as compared to vehicle-treated hCMED/D3 cells. It is of interest that the reduced transendothelial migration of monocytes across treated ECs coincided with a decreased mRNA expression of the leukocyte adhesion molecule VCAM-1 (Figure 3c) and increased expression of the cell–cell junction protein VE-cadherin (Figure 3d), suggesting that S1P_5_ agonism reduced the inflammatory status of the brain endothelium.

### S1P5 is essential for the brain EC barrier phenotype

**Figure 4:**
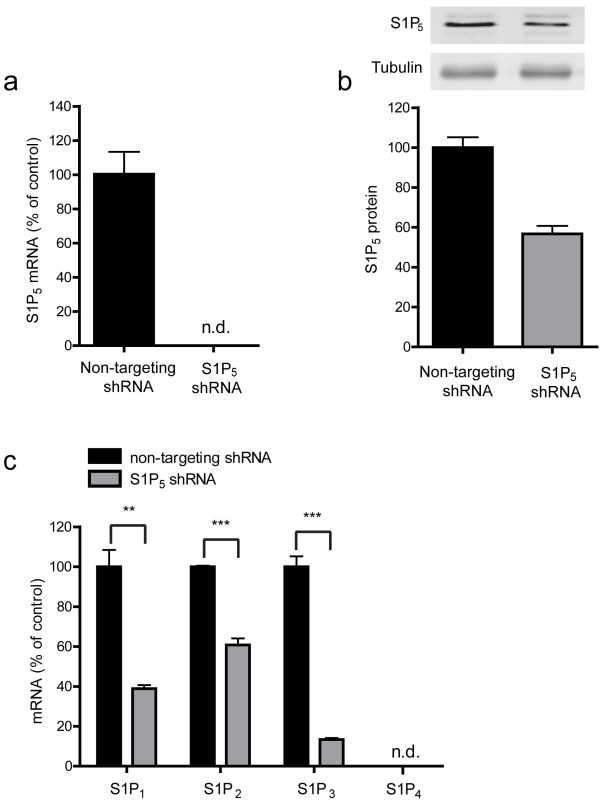
**S1P**_**5**_**knockdown and downstream effects on endothelial S1P receptor expression.** (**a**) A lentiviral shRNA technique was used to knockdown the S1P_5_. Treatment of the brain ECs with specific shRNA clone E2 resulted in a non-detectable level of S1P_5_ RNA as measured by qPCR. S1P_5_ RNA expression levels in control cells were set at 100%. (**b**) Western blot analysis shows a 43.1% ± 2.12% reduction in S1P_5_ protein levels when compared to control cells. (**c**) S1P receptor characterization in S1P_5_ knockdown cells show reduction in RNA expression levels of all S1P receptors where S1P_1_ is reduced by 61% (non-targeting control:100.0% ± 8.5, S1P_5_ knockdown: 38.9% ± 1.8), S1P_2_ by 39% (non-targeting control: 100.0% ± 0.6, S1P_5_ knockdown: 60.8% ± 3.3), and S1P_3_ by 87% (non-targeting control: 100.0% ± 5.3; S1P_5_ knockdown 13.3% ± 0.8). Again, S1P_4_ remains undetectable (n.d.) Values represent the mean ± SEM of n = 3. Statistical significance (*t*-test) is indicated with asterisks: **P < 0.002 and ***P < 0.001.

To further elucidate the mechanism of the S1P_5_ at the brain endothelium, we generated a brain EC line (hCMED/D3 subclone E2) with reduced expression of S1P_5_. Transduction of brain ECs with lentiviruses expressing a S1P_5_-specific shRNAs reduced the expression of S1P_5_ as determined by qPCR to undetectable levels (Figure 4a). Protein levels of S1P_5_ were reduced by 43% (Figure 4b). Of note, S1P_5_ knockdown cells also displayed reduced expression of other S1P receptors including S1P_1,_ S1P_2_ and S1P_3_ (Figure 4c). These findings suggest that S1P_5_ regulates the expression of other S1P receptors.

**Figure 5:**
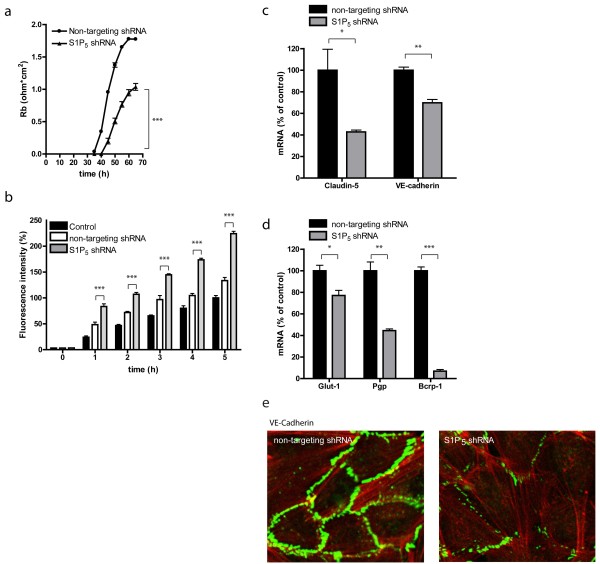
**S1P**_**5**_**regulates several key features of barrier endothelium.** (**a**) ECIS was used to measure the impedance of the brain endothelial monolayer. S1P_5_ knockdown cells (1.0 ohm.cm^2^ ± 0.1) have over 40% reduction in TEER compared to mock cells (1.8 ohm.cm^2^ ± 0.02). Rb values represent the mean ± SEM of four individual experiments. (**b**) Paracellular permeability was studied by passive leakage of FITC-dextran molecules (70kD). S1P_5_ knockdown cells (224.1% ± 4.6 at 5 h) were more permeable FITC-dextrans in time compared to control and mock transduced ECs (133.4% ± 6.1 at 5 h). Fluorescence intensity values represent mean ± SEM of four individual experiments with hCMEC/D3. (**c**) qPCR analysis of tight junction-associated proteins in S1P_5_ knockdown cells compared to control cells revealed a 57% decrease in claudin-5 expression (non-targeting control: 100.0% ± 19.4; S1P_5_ knockdown 42. 8% ± 1.8, n = 3) and a 30% decrease in VE-cadherin expression (non-targeting control: 100.0% ± 2.9; S1P_5_ knockdown 69.8% ± 3.2, n = 3). (**d**) qPCR analysis of transporters expressed at the BBB revealed a 23% decrease in GLUT-1 expression (non-targeting control: 100.0% ± 5.1; S1P_5_ knockdown 77.1% ± 4.7, n = 3), 55% decrease in Pgp expression (non-targeting control: 100.0% ± 8.1; S1P_5_ knockdown 44.5% ± 1.60, n = 3), and 93% decrease in BCRP1 expression (non-targeting control: 100.0% ± 3.5; S1P_5_ knockdown 7.0% ± 1.4, n = 3). (**e**) VE-cadherin localization was also studied by immunocytochemistry. Reduced junctional VE-cadherin localization was observed in S1P_5_ knockdown cells compared to control cells (VE-cadherin, green and F-actin, red). Statistical significance (*t*-test) is indicated with asterisks: *P < 0.05, **P < 0.002 and ***P < 0.001.

Next, ECIS analysis was performed to determine the function of S1P_5_ in brain EC barrier formation. Interestingly, S1P_5_ knockdown cells revealed reduced barrier integrity compared to control cells (Figure 5a). To further delineate function of S1P_5_ on brain endothelial function, we studied various other properties that are associated with BBB functioning. First, S1P_5_ knockdown cells were more permeable to FITC-dextrans compared to control cells (Figure 5b), illustrating enhanced leakiness of the brain endothelial layer. Second, and in accordance with these data, S1P_5_ knockdown ECs displayed reduced expression levels of tight junction and adherent junction-associated proteins such as claudin-5 and VE-cadherin (Figure 5c). Our analyses further show that key BBB-associated transporter proteins like the nutrient transporter for glucose (GLUT-1), and the ATP-binding cassette (ABC) transporters P-glycoprotein (Pgp) and breast cancer resistant protein-1 (BCRP-1) were significantly decreased (Figure 5d) upon S1P_5_ knockdown. Consistent with the observed reduced VE-cadherin expression level in S1P_5_ knockdown cells, we found impaired VE-cadherin protein localization to the junctions in the S1P_5_ knockdown cells (Figure 5e). Together, these data show that S1P_5_ has a crucial role in the maintenance of the barrier phenotype in brain ECs.

### S1P_5_ contributes to the immune quiescence state of the brain EC barrier

Under normal conditions, the brain endothelium promotes immunoquiescence of the brain by a low expression of cell adhesion molecules and undetectable detection of the production of proinflammatory cytokines and chemokines, thereby limiting attraction and subsequent transendothelial migration of leukocytes.

**Figure 6:**
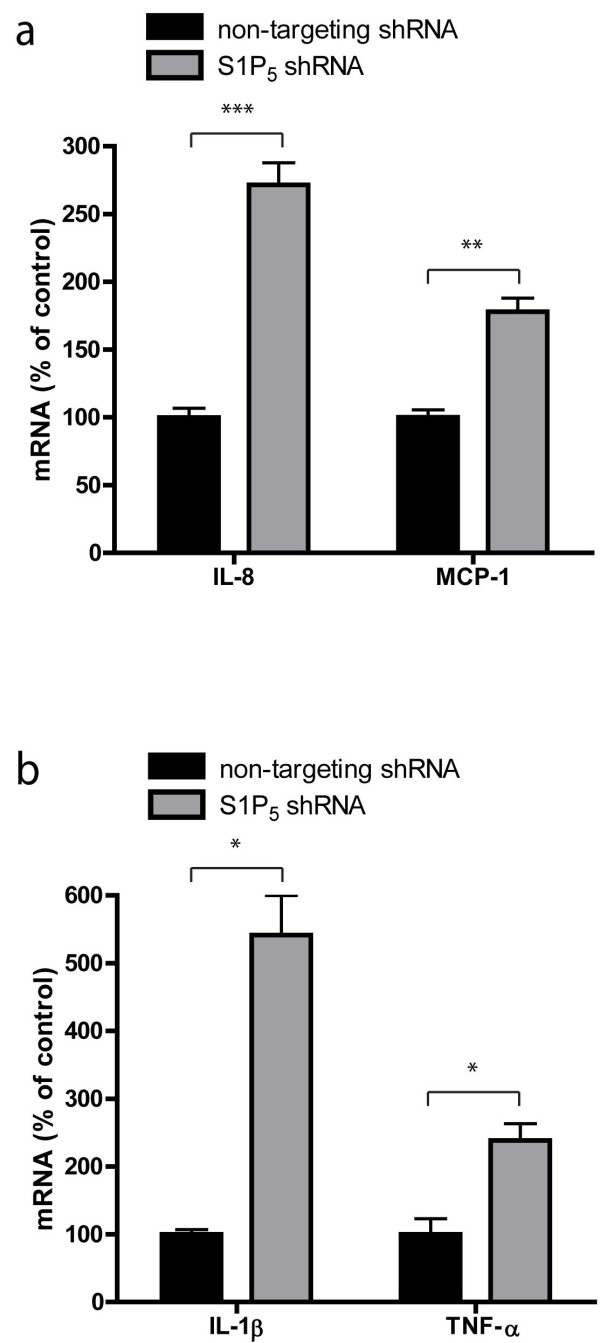
**S1P**_**5**_**knockdown abolishes the immune quiescent state of the brain endothelial cell barrier.** (**a**) RNA expression of proinflammatory cytokines and chemokines were assayed by qPCR. RNA expression of the chemokines MCP-1 and IL-8 are increased by 78% (control: 100.0% ± 5.6; S1P_5_ knockdown 178.1% ± 9. 9, n = 3) and 171% (control: 100.0% ± 6.7; S1P_5_ knockdown 271.4% ± 16.3, n = 3) respectively in S1P_5_-deficient cells compared to mock cells. (**b**) Levels of the endothelial cytokines were increased upon S1P_5_ deficiency, IL-1β by 442% (control: 100.0% ± 7.1; S1P_5_ knockdown 541.5% ± 58. 5, n = 3) and TNF-α by 138% (control: 100.0% ± 22.9; S1P_5_ knockdown 238.4% ± 24.7, n = 3). Statistical significance (*t*-test) is indicated with asterisks: *P < 0.05, **P < 0.002 and ***P < 0.001.

To investigate the role of S1P_5_ in the inflammatory status of the brain endothelium, we studied gene expression levels of cytokines and chemokines known to be involved in the neuroinflammatory attack such as the chemokines monocyte chemoattractant protein-1 (MCP-1: CCL2) and interleukin-8 (IL-8) and the proinflammatory cytokines interleukin-1β (IL-1β), and tumor necrosis factor-α (TNF-α). Upon S1P_5_ knockdown, mRNA levels of MCP-1 and IL-8 as well as the levels of TNF- α and IL-1 β increased dramatically (Figure 6a,b).

**Figure 7:**
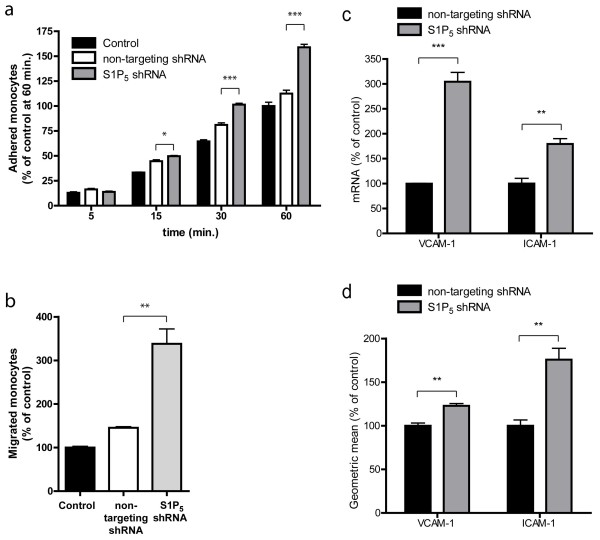
**S1P**_**5**_**knockdown increases monocyte transmigration and adhesion.** (**a**) Adhesion of primary human monocytes to hCMEC/D3, mock, and S1P_5_ cells was assayed in time. A significant increase in monocyte adhesion to S1P5 knockdown cells was already observed after 15 minutes (non-targeting control: 112.5% ± 3.4; S1P_5_ knockdown 159.0% ± 3.0 at 60 minutes, n = 6). (**b**) Monocyte migration across confluent monolayers of hCMEC/D3, mock, and S1P5 knockdown cells was assayed by time-lapse microscopy. A strong increase in monocyte migration of 193% was observed across S1P5 knockdown cells compared to mock cells (non-targeting control: 145.5% ± 2.6; S1P_5_ knockdown: 338.1% ± 34.2, n = 3). (**c**) VCAM-1 and ICAM-1 gene expression levels in mock and S1P_5_ knockdown cells were studied by qPCR. VCAM-1 gene expression increased 204% (non-targeting control: 100.0% ± 0.5; S1P_5_ knockdown 304.4 ± 18.8, n = 3) and ICAM-1 gene expression increased 80% (non-targeting control: 100.0% ± 10.6; S1P_5_ knockdown 179.6% ± 10.8, n = 3) in S1P_5_ knockdown cells compared to mock cells. (**d**) FACS analysis of ICAM-1 and VCAM-1 expression by mock cells and S1P_5_ knockdown cells demonstrated a 76% (non-targeting control: 100.0% ± 6.6; S1P_5_ knockdown: 175.8% ± 13.1, n = 3) increase in ICAM-1 protein expression and a 23% (non-targeting control: 100.0% ± 3.1; S1P_5_ knockdown: 122.8% ± 2.7, n = 3) increase in VCAM-1 protein expression by S1P_5_ knockdown cells. Statistical significance (*t*-test) is indicated with asterisks: *P < 0.05, **P < 0.005 and ***P < 0.001.

Interestingly, our results showed that activation of S1P receptors, and in particular S1P_5_, reduced the expression of adhesion molecules and enhanced the capacity of brain ECs to prevent monocyte passage (Figure 3). We next determined the function of S1P_5_ in these distinct aspects of the BBB. As expected, knockdown of the S1P_5_ in brain endothelium enhanced the adhesion of monocytes to, and migration of, monocytes through the endothelium when compared to mock and control hCMEC/D3 cells (Figure 7a,b).

To reveal the underlying mechanism of this enhanced monocyte-endothelial interaction we next determined the expression levels of adhesion molecules ICAM-1 and VCAM-1 which are both known to be involved in the adhesion and migration of monocytes [35]. Our results show that both gene and protein expression levels of VCAM-1 and ICAM-1 were significantly increased in S1P_5_ knockdown cells compared to control cells (Figure 7c,d). Together, these findings indicate that steady-state presence of cell surface S1P_5_ strongly contributes to the immune quiescence state of the BBB.

### The inflammatory phenotype is partly mediated by NF-κB activation

**Figure 8:**
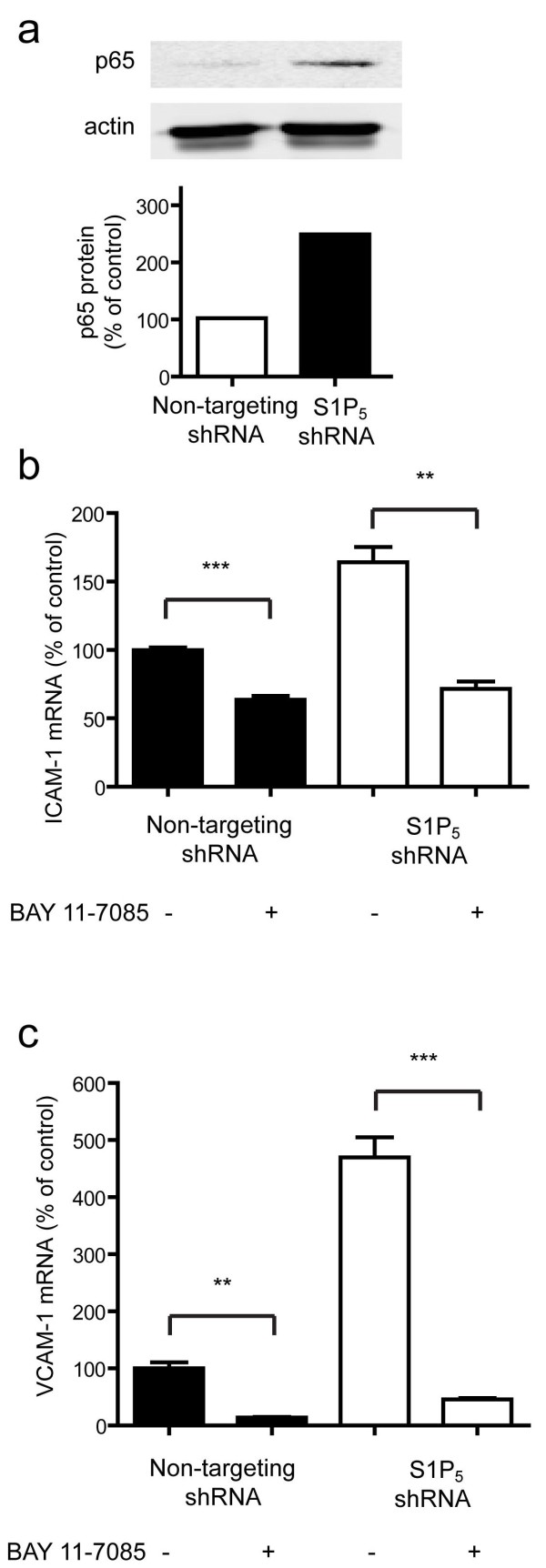
**S1P**_**5**_**mediates immune quiescence of brain endothelial cells by NF-κB.** (**a**) Protein content of p65 in S1P_5_ knockdown and control cells was assessed by western blot. The protein expression of control cells was set as 100%. The S1P_5_ knockdown cells show an increased expression of about 250% compared to control. The expression of S1P_5_ was corrected for actin. (**b**) Mock cells and S1P_5_ knockdown cells were left unexposed or were exposed to NF-κB inhibitor BAY 11–7085 and ICAM-1 gene expression levels were determined by qPCR. Upon exposure to BAY 11–7085 ICAM-1 RNA expression in S1P_5_ knockdown cells decreased to approximately the same level as in BAY 11–7085 exposed mock cells. The ratio of reduction in ICAM-1 gene expression by inhibition of NF-κB in S1P_5_ knockdown cells increased 2.3-fold compared to 1.6-fold in mock cells. (**c**) Mock cells and S1P_5_ knockdown cells were left unexposed or were exposed to NF-κB inhibitor BAY 11–7085 and VCAM-1 gene expression levels were determined by qPCR. Upon exposure to BAY 11–7085 VCAM-1 RNA expression in S1P_5_ knockdown cells decreased to approximately the same level as in BAY 11–7085 exposed mock cells. The ratio of reduction in VCAM-1 gene expression by inhibition of NF-κB in S1P_5_ knockdown cells is increased to 10.3-fold compared to 7.3-fold in mock cells. Data are expressed as the mean ± SEM (n = 3). Statistical significance (*t*-test) is indicated with asterisks: *P < 0.05, **P < 0.002 and ***P < 0.001.

As both ICAM-1 and VCAM-1 expression are under the control of the NF-κB signaling pathway, we studied whether brain endothelial S1P_5_ exert their anti-inflammatory control through NF-κB. First we determined the regulation of the total p65 subunit of NF-κB protein content by western blot. As shown in Figure 8a, the S1P_5_ knockdown cells have an increased basal expression of the p65 subunit of NF-κB when compared to control cells. This increased NF-κB content, which might underlie the increased expression of adhesion molecules, strongly suggests that S1P_5_ modulation is involved in immune quiescence of brain ECs mediated by inhibition of NF-κB. The selective NF-κB inhibitor Bay 11–7085 significantly reduced the gene expression levels of both ICAM-1 and VCAM-1 (Figure 8b,c). Interestingly, addition of Bay-11–7085 to S1P_5_ knockdown resulted in relative decreased gene expression levels of ICAM-1 and VCAM-1 compared to the mock control cells. To reveal the effect of NF-κB in the S1P_5_ knockdown cells, the ratio of reduced expression of ICAM-1 and VCAM-1 was calculated. It was shown that the ratio of reduction in ICAM-1 gene expression by inhibition of NF-κB in S1P_5_ knockdown cells was increased over 2-fold compared to 1.6-fold in mock cells. Identically, the ratio of reduction in VCAM-1 gene expression by inhibition of NF-κB in S1P_5_ knockdown cells is increased to over 10-fold compared to 7.3-fold in mock cells.

## Discussion

Our present study demonstrates the constitutive expression of the S1P receptor S1P_5_ by brain ECs, constituting the BBB in human brain and its involvement in both integrity and regulation of the inflammatory status of the brain endothelium. We have delineated a role for S1P_5_ in the induction of specific BBB properties such as low paracellular permeability and the expression of key brain endothelial proteins such as tight junction and specific ATP binding cassette and glucose transporter molecules. We have assessed the potential therapeutic improvements upon pharmacological modulation of S1P_5_ receptor activity in gaining barrier function. Moreover, the lack of S1P_5_ provoked a proinflammatory status of brain endothelium as shown by enhanced transendothelial migration of monocytes and increased production of proinflammatory molecules and adhesion molecules for leukocytes, a process which was mediated by NF-κB activation.

Within our findings, we showed the non-selective S1P receptor agonist FTY720P and a selective S1P_5_ agonist both improve brain endothelial function through the increase of the paracellular resistance in the immortalized human brain EC line, hCMEC/D3. This cell line reflects the key features of primary brain ECs such as high TEER, expression of tight junctions and transporter molecules. Our results are in line with previous reports on the endothelial barrier enhancing effect of FTY720P on pulmonary ECs, human microvascular ECs, and human umbilical vein ECs [22,36-38]. However, we are the first to show that selective activation of S1P_5_ induces a significant increase in paracellular resistance of brain ECs. Moreover, in concordance with ECs exposed to FTY720P, brain endothelium exposed to a selective S1P_5_ agonistic compound prevented the transendothelial passage of monocytes in a similar fashion [31]. Enhanced endothelial barrier integrity is probably the result of the enhanced expression of tight junction and adherent junction proteins and their localization at the cell–cell contacts, as was shown in this study. To date, there is emerging evidence that S1P_1_ and S1P_3_ play an important role in the maintenance of endothelial function. For instance, it has been described that the assembly of junction proteins to the cell–cell junctions is dependent on S1P_1_ and S1P_3_-induced signaling pathways, involving the small GTP-ases Rac and Rho [39]. Strikingly, in our S1P_5_-deficient brain ECs a significant reduction of S1P_1_ and S1P_3_ was detected, indicating that S1P_5_ is involved in the expression of these receptors. Given the importance of S1P_1_ and S1P_3_ in the maintenance of vascular function, the reduced barrier function of the brain endothelium due to the lack of S1P_5_ may also be in part caused by the lack of S1P_1_ and S1P_3_. Together, our findings point to an important function of S1P_5_ in the regulation of the barrier phenotype of brain ECs.

Besides improving barrier function, the current report shows that targeting of S1P_5_ is important for maintenance of the immunoquiescent state of brain ECs. First, our results reveal a prominent role for S1P_5_ in maintaining low expression levels of leukocyte adhesion molecules, including VCAM-1 and ICAM-1, and several important proinflammatory cytokines and chemokines, such as TNF-α and MCP-1. In addition, S1P_5_ activation limits monocyte adhesion to and migration over the brain endothelial barrier, which is an initial step in the formation of new MS lesions. Therefore, our findings are of potential interest for future treatment of neuroinflammatory disorders that are marked by a cellular migration across the vessel wall. Modulation of the S1P receptors through FTY720P was shown to be protective in various experimental animal models with neuroinflammation in the brain [19,40,41]. Moreover, in other vascular disorders, such as atherosclerosis, FTY720P exerts protective effects by dampening ongoing inflammatory processes in the vascular smooth muscle cells. In the validated experimental animal model for MS, experimental allergic encephalomyelitis FTY720P was either given as a prophylactic, therapeutic or as a rescue agent. In these animals, FTY720P treatment resulted in a delayed onset of disease and reduced disease severity through the induction of lymphopenia. Besides its general immunosuppressive effect, FTY720P treatment also reduced the expression of vascular adhesion molecules and cytokines in the spinal cord of treated animals [19]. Moreover, in models of brain ischemia FTY720P was able to reduce infarct size and could, in transient focal cerebral ischemia, positively regulate neurological deficits. Observed beneficial effects were associated with a reduction in activated neutrophil and microglia/macrophage cell numbers and, in line with our results, also with reduced numbers of ICAM-1 positive blood vessels [41]. However, these studies did not investigate the role of endothelial S1P signaling, which may play a crucial role in the early onset of disease [40]. Several *in vitro* studies support the notion that S1P signaling is relevant in ECs because it was shown that S1P or/and FTY720P limit leukocyte adhesion and transmigration into the vessel wall in the early atherosclerosis pathology. In addition, S1P_1_ modulation resulted in reduced endothelial production of TNF-α-induced production of proinflammatory chemokines [42-44]. Given that both S1P and FTY720P are general modulators of all S1P receptors, it remains unclear which specific receptor is involved in these processes. More specifically, it is unclear what the role of the brain-specific S1P_5_ receptor is in these processes. Our study clearly shows that S1P_5_ receptors might play a crucial role in barrier formation and immunoquiescence. Although this study utilized a selective S1P_5_ receptor agonist, it is important to identify new more potent and more selective S1P_5_ tools to fully elucidate the mechanism by which S1P_5_ might exert these effects.

We are also the first to show that NF-κB underlies the anti-inflammatory activity of endothelial S1P_5_. To date, no data exist on the downstream signaling pathways in ECs upon activation of S1P_5_. It has been shown that incubation of mouse aortas from type 1 non-obese diabetic mice with S1P reduces VCAM expression and monocyte adhesion to the endothelium. The authors also show that S1P_1_ is the receptor that mediates this anti-inflammatory response to S1P, which is related to the inhibition of NFkB translocation to the nucleus. In accordance with these findings, our results suggest an anti-inflammatory role for S1P_5_ in brain ECs. Furthermore, in Chinese Hamster Ovary-K1 cells it was demonstrated that S1P_5_ expression results in inhibition of extracellular signal-regulated kinase (ERK) activity [45]. Since ERK activity is a potent regulator of NFκB-dependent inflammatory responses, this pathway may be responsible for the observed increase in NF-κB driven of ICAM-1 and VCAM expression in S1P_5_ knockdown brain ECs.

## Conclusion

Together, our novel and provocative findings emphasize the importance of S1P signaling and in particular S1P_5_ signaling during health and disease. We demonstrate that endogenous endothelial S1P_5_ represents an essential receptor for optimal BBB function by regulating different aspects of the BBB, including the expression of cell–cell junction proteins and efflux pumps. Importantly, we demonstrate that S1P_5_ is essential for maintenance of the immune quiescent state of brain ECs and that this is in part mediated by NF-κB. Our data also reveal the therapeutic potential of S1P_5_ agonism during neuroinflammation as S1P_5_ specifically limits neurovascular inflammation and transendothelial leukocyte infiltration.

## Abbreviations

ABC, ATP-binding cassette; BBB, Blood-brain barrier; BCRP, Breast cancer resistant protein-1; bFgF, Basic fibroblast growth factor; BSA, Bovine serum albumin; CNS, Central nervous system; DMSO, Dimethyl sulfoxide; EC, Endothelial cell; ECIS, Electrical cell-substrate impedance sensing; EDG, Endothelial differentiation gene; ERK, Extracellular signal-regulated kinase; FCS, Fetal calf serum; GLUT-1, Glucose transporter-1; GPCR, G-protein-coupled receptor; ICAM-1, Intercellular adhesion molecule-1; IL-1β, Interleukin-1β; IL-8, Interleukin-8; IRdye, Infrared dye; MCP-1, Monocyte chemoattractant protein-1; MS, Multiple sclerosis; NFκB, Nuclear factor kappa-light-chain-enhancer of activated B cells; NGS, Normal goat serum; PBS, Phosphate-buffered saline; Pgp, P-glycoprotein; qPCR, quantitative PCR; S1P, Sphingosine 1-phosphate; SDS, Sodium dodecyl sulfate; shRNA, short hairpin RNA; TEER, Transendothelial electrical resistance; TNF-α, Tumor necrosis factor-α; VCAM-1, Vascular cell adhesion molecule-1.

## Competing interests

The authors declare that they have no competing interests.

## Authors’ contributions

RvD carried out the experiments, analyzed the data and drafted the manuscript. MALP carried out the NFκB analyses. GK performed the transmigration studies. KL, BvhH and SvdP did the culture work and participated in the qPCR analyses. DG provided the shRNA construct. JvH and PvdV were responsible for the human brain tissues and immunohistochemical analyses. EvdK and ER participated in design and coordination, provided useful advice and reviewed the manuscript. AR and HEdV participated in the design and coordination, and wrote and reviewed the manuscript. All authors read and approved the final manuscript.
